# Aerospace Technology Improves Fermentation Potential of Microorganisms

**DOI:** 10.3389/fmicb.2022.896556

**Published:** 2022-04-29

**Authors:** Yan Chi, Xuejiang Wang, Feng Li, Zhikai Zhang, Peiwen Tan

**Affiliations:** ^1^Wuzhoufeng Agricultural Science and Technology Co., Ltd., Yantai, China; ^2^Department of Computer Science, University of California, Irvine, Irvine, CA, United States

**Keywords:** extreme environment, microorganism, production improvement, fermentation improvement, genetic mutant, aerospace technology

## Abstract

It is highly possible to obtain high-quality microbial products in appreciable amounts, as aerospace technology is advancing continuously. Genome-wide genetic variations in microorganisms can be triggered by space microgravity and radiation. Mutation rate is high, mutant range is wide, and final mutant character is stable. Therefore, space microorganism breeding is growing to be a new and promising area in microbial science and has greatly propelled the development of fermentation technology. Numerous studies have discovered the following improvements of fermentation potential in microorganisms after exposure to space: (1) reduction in fermentation cycle and increase in growth rate; (2) improvement of mixed fermentation species; (3) increase in bacterial conjugation efficiency and motility; (4) improvement of the bioactivity of various key enzymes and product quality; (5) enhancement of multiple adverse stress resistance; (6) improvement of fermentation metabolites, flavor, appearance, and stability. Aerospace fermentation technology predominantly contributes to bioprocessing in a microgravity environment. Unlike terrestrial fermentation, aerospace fermentation keeps cells suspended in the fluid medium without significant shear forces. Space radiation and microgravity have physical, chemical, and biological effects on mutant microorganisms by causing alternation in fluid dynamics and genome, transcriptome, proteome, and metabolome levels.

## Introduction

Microorganism fermentation is the most prominent and rapidly growing segment of biological sciences, and fermentation of microbes and their products are closely associated with agriculture and the food and pharmaceutical industries (Kalsoom et al., 2020). However, there are some challenges for industrial fermentation, including limited biomass, time-consuming to reach steady-state and low cell densities (Yang and Sha, 2019), and low yields and nutri. Electrical energy is mainly used for industrial fermentation. However, electrical fields may affect the fermentation bioprocess by altering its micronutrients (Gavahian and Tiwari, 2020). Semi-solid and submerged fermentations have been widely conducted in industries but with low-level yields and time spent because of terrestrial gravity. The space’s extreme environment, with high-level radiation and microgravity, may address these important issues *via* wide-range mutants.

**FIGURE 1:**
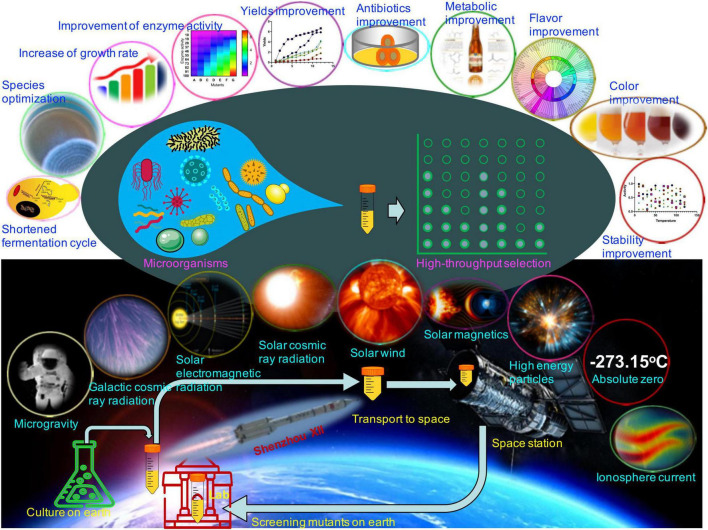
Possible effects of the space’s extreme environment on the fermentation potential of microorganisms. The space’s environment mainly includes microgravity, space radiation (galactic cosmic ray, electromagnetic waves, solar cosmic ray, solar wind, and solar magnetic and/or high energetic particles), ionosphere current, and a degree above absolute zero. Most microorganism mutants may be induced with the improvement of fermentation potentials *via* the space’s extreme environment, including species, growth rate, enzyme activities, product yields, antibiotic activities, metabolite composition, fermentation flavor and color, and strain stability (Supplementary Table 1).

The space’s extreme environment, with a temperature above absolute zero degrees, mainly includes microgravity, space radiation (in the form of rays, electromagnetic waves, and/or high energic particles), the ionosphere ionized by solar and cosmic radiations, ultra-vacuum, etc. (Figure 1). Space microgravity is defined as gravity less than 10^–4^ G (1 G is defined as 9.8 m/s^2^) in the space’s environment. Under the space environment, mutant DNA occurs at a global chromosomal level because of the deletion, replacement, or insertion of bases, which is higher than on the earth (Mulkey, 2010) and improves the fermentation potentials of microorganisms, such as *Lactobacillus acidophilus* (Shao et al., 2017), *Saccharomyces cerevisiae* (Liu et al., 2008), and *Bacillus subtilis* (Nicholson and Ricco, 2019).

Since September 21, 1992, China Manned Space Engineering has been burgeoning. In the past 30 years, it has attained significant success in aerospace technology, including the launch of a series of Shenzhou spacecraft. China started space microorganism experiments in the Shenzhou I spacecraft on November 20, 1999 (Fang et al., 2005), and has accumulated a visual experience in effects of the space’s extreme environment on microorganisms (Figure 1 and Supplementary Table 1). In September 2010, China officially launched its space station program, and space microorganism sciences have been steadily developing ever since (Long, 2016). On November 1, 2011, the Shenzhou VIII spacecraft began a large-scale space microbiology experiment equipped with 15 microorganisms (Su et al., 2013). The study revealed changes in bacterial invasion, antibiotic resistance, and environmental adaptation. The mechanisms may be caused by various factors, including genome, transcriptome, proteome, and metabolome. On the same date, engineered bacterial strains with recombinant human interferon a1b were launched into the space station. Five mutant engineered bacterial strains showed significantly higher production of recombinant human interferon a1b and one strain with twofold increase in antibiotic activities (Wang et al., 2014). The mutant tetrodotoxin strains *via* spatial mutagenesis can be used for industrial production of toxins. After purification of toxins, they are mainly used for detoxification and effectively reduce the relapse rate of addicts (China patent no. CN103160454B) (Mulkey, 2010). On June 11, 2013, *Lysobacter enzymogenes* was trained in the space environment *via* the Shenzhou X spacecraft (Liu, 2017). The mutants showed increase in the production of endoproteinase Lys-C by up to 40.2% with perfect stability. On October 17, 2016, *Acinetobacter baumannii* was trained in the space environment *via* the Shenzhou XI spacecraft. The ability for biofilm formation of the mutant strain was reduced (Zhao et al., 2019).

The effect of the space environment on production of antibiotic actinomycin D by *Streptomyces plicatus* was tested in US Space Shuttle STS-80. The space flight reduced the number of cells in CFU/ml of *S. plicatus* and increased the productivity of actinomycin D (Lam et al., 2002). Deletion of the ribosomal protein gene in the yeast *Saccharomyces cerevisiae* was detected after flight in the Russian space station, suggesting that space radiation containing high-linear energy transfer causes deletion-type mutants (Fukuda et al., 2000). In another study, three fungal species, *Aspergillus sydowii*, *Penicillium palitans*, and *Rhodotorula mucilaginosa*, grew in the Japanese Space Station KIBO for 7 years and the fungi are still increasing and expanding over time (Satoh et al., 2021).

Whole-genome sequencing and bioinformatics indicated changes at the genome, transcriptome, proteome, and metabolome levels, which contribute to phenotypic changes of mutant strains (Kimura et al., 2006; Sakai et al., 2018). Most mutants may be induced with improvement of fermentation potentials *via* the space’s extreme environment. Mutants can be screened *via* high-throughput techniques in a laboratory on Earth and can be found with improvement in fermented microorganisms, including (1) shortened fermentation cycle and increased growth rate because of decreasing lag phase and prolonging exponential phase *via* upregulation of DNA replicon gene (*srmB*) and repression of nucleoside metabolism genes (dfp, pyrD, and spoT) (Arunasri et al., 2013; Senatore et al., 2018); (2) optimization of fermented mixed species (Zongzhou and Yaping, 2009); (3) increase in bacterial conjugation efficiency and motility by stimulation of plasmid transfer (Beuls et al., 2009) and gene regulation of flagellar synthesis and function and/or taxis (Acres et al., 2021); motility induces three-dimensional transitions of bacterial monolayers (Takatori and Mandadapu, 2020); (4) improvement of key enzyme bioactivity and product quality (Zhang et al., 2015); and (5) improvement of metabolite production, flavor, appearance, and stability (Figure 1 and Supplementary Table 1; Bin et al., 2015; Senatore et al., 2020). Therefore, aerospace technology provides an unprecedented platform for exploring microorganism’s utilization systems.

## Mechanisms for the Physical, Chemical, and Biological Effects of Space Microgravity and Radiation on the Fermentation Potential of Microorganisms

Space microgravity induces mutant microorganisms. Microgravity can affect physical and chemical environmental parameters and induce mutant strains. Kanglemycin C is an immunosuppressant produced by *Nocardia mediterranei var. kanglensis* but with limited yields. Space flight can induce Kanglemycin C-producing mutant strains with high-level products (Zhou et al., 2006). The marine bacterium *Vibrio fischeri* was tested during long-duration spaceflight. The results showed that rodA was depleted, but that impacts on symbiont genes were minimal under microgravity (Burgos et al., 2020). On the other hand, microgravity may increase bacterial conjugation efficiency by stimulating plasmid transfer (De Boever et al., 2007). Some phenotype changes of space microorganisms may be caused by alternation in the metabolic pathway and fluid dynamics.

**FIGURE 2:**
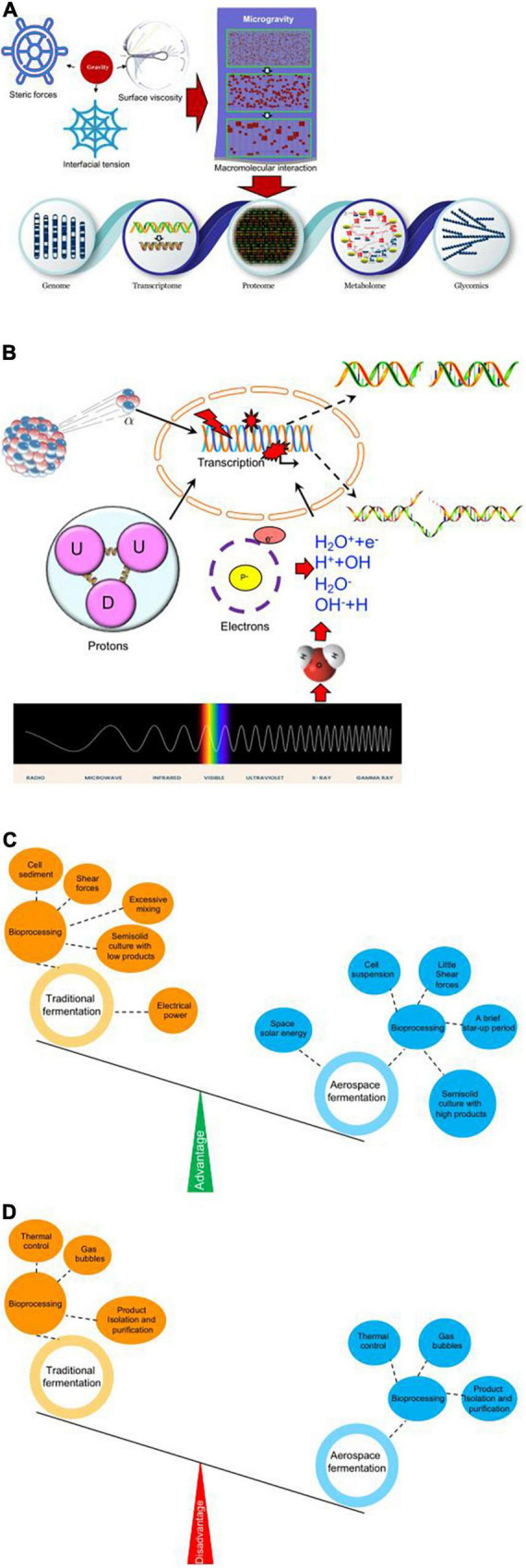
Possible functional mechanisms for space microbiology technology. **(A)** Possible physical and molecular mechanisms for space microgravity-inducing mutant microorganisms. **(B)** Physical, chemical, and biological effects of space radiation. X-ray and alpha-particles induce DNA double-strand break and UV irradiation and gamma irradiation induce DNA single-strand breaks. **(C)** Advantages of space fermentation technology when compared with terrestrial fermentation technology. **(D)** Disadvantages of space fermentation technology when compared with terrestrial fermentation technology.

Although the space’s microgravity can induce microbe mutants at the genome, transcriptome, proteome, and metabolome levels, the exact mechanism for microgravity-inducing mutants remains unclear. Molecular weight affects steric forces, interfacial tension, and surface viscosity, which all have an influence on molecule distribution (Bąk and Podgórska, 2016). Thus, microgravity will change these parameters, which possibly affect protein crystallization (Durbin and Feher, 1996). Protein crystallization is found to be easily formed in the space’s microgravity environment (Scott and Vonortas, 2021). Therefore, we propose that microgravity will affect the physical environment of biological molecules and their interaction. Changes in macromolecules interaction will lead to changes in genome structure, transcriptome, proteome, metabolome, and glycomics (Figure 2A).

## Space Radiation Induces Mutant Microorganisms

Space is filled with high-energy particles (including alpha, protons, electrons, and neutrons) and electromagnetic waves (gamma rays and X-rays), which can cause high-level mutation of DNAs and proteins. Space radiation causes changes in spore survival and rifampicin resistance in Bacillus species by inducing amino acid mutants at sites Q469L, A478V, and H482P/Y (Moeller et al., 2010). An interplay between microgravity and space radiation can induce DNA strand breaks, chromosome abnormalities, micronucleus formation, or various mutants (Moreno-Villanueva et al., 2017). Some forms of radiation affect the ability for microbial biofilm formation by surface barrier discharge (Salgado et al., 2021). *S. cerevisiae* irradiated with gamma rays had genome-wide variants because of DNA strand break (Chan et al., 2007).

Space radiation has physical, chemical, and biological effects on mutant microorganisms with various rays and particles. X-ray and alpha-particles induce DNA double-strand break (Newman et al., 1997) and UV- and gamma-irradiation-induced DNA single-strand breaks in microorganisms (Figure 2B; Lankinen et al., 1996). An electron particle or X-ray triggers H_2_O molecules to ionize and disrupt, and produce low-energy electrons and OH-radicals, which contribute to DNA strand break (Figure 2B).

## Comparison of Terrestrial Fermentation and Space Fermentation Technology

Aerospace fermentation technology predominantly contributes to bioprocessing with its unique space microgravity environment. Unlike terrestrial fermentation, aerospace fermentation keeps cells suspended in the fluid medium without significant shear forces, which are often caused by stirred terrestrial systems (Figure 2C). A space fermentation device, clinostat, provides a method of keeping cell movement in liquid without introducing excessive mixing *via* the rotational velocity of vessel’s inner walls (Topolski, 2021). Meanwhile, cell sedimentation can be prevented *via* microgravity (Figure 2C). On the other hand, semi-solid culture is often limited to low-level target products in a terrestrial lab because of gravity, which can be overcome *via* space fermentation technology (Figure 2C). Finally, energy can be saved during space fermentation *via* space solar energy, while electrical power is a predominant way to supply energy during terrestrial fermentation (Figure 2C). Certainly, there are some disadvantages for space fermentation technology when compared with terrestrial fermentation; there are some difficulties in dealing with thermal control, gas bubbles, and product isolation and purification because of the lack of gravity (Figure 2D).

## Discussion

Space is a special environment consisting of microgravity and strong radiation, and plays an important role in producing various mutant microorganisms with health-promoting properties or improved fermentation potentials. Important research results and practical applications of microorganisms with the help of aerospace technology have been achieved in microbial pharmaceuticals, microbial fertilizers, and wine-making fields. Mutant microorganisms caused by aerospace technology have broad research prospects and research value.

To improve the quality of fermentation products, the quality of yeast is very crucial. In the brewing process, yeast plays an important role during the conversion of sugar into ethanol, and this process will affect the quality and yield of wine. To get better-performing brewing functional flora, creation of mutant strains at a genome-wide level is available *via* aerospace technology. The wine and beer industries have been dominated by *Saccharomyces cerevisiae* in the world (Peris et al., 2018). *S. cerevisiae* is “indispensable” as a contributor to the flavor-active metabolite profile and aroma-active compounds of beers (Kutyna and Borneman, 2018). Beer yeast mutants (*S. cerevisiae* HT-1, HT-2, and HT-3) were obtained from the Shenzhou VIII spacecraft and produced more active metabolites that are beneficial to human health and further improve product quality, flavor, and appearance (Bin et al., 2015). The fermentation beer was separated from the yeast sediment by centrifugation.

Aerospace biotechnology opens a way for effectively cultivating new varieties and special germplasm resources and has a bright future ahead. With the improvement of fermentation products, there is a boom in modern industry and agriculture. With the continuous development of world spacecraft, space methods will be applied to various areas, pushing for more reliable studies on space microorganisms. However, there are still some challenges for space microorganism research. Apparently, there is still a lack of effective methods to avoid the generation of harmful mutant microbes, and some of them may be deadly. Post-spaceflight lab screening lacks methods for controlling the direction of mutagenesis, and more mutagenesis pathways need to be further explored and investigated. Most human pathogenic isolates from space stations have been found to be multidrug-resistant, such as sulfamethoxazole, erythromycin, and ampicillin, which will cause adilemma in the antibiotic industry.

## Author Contributions

YC and XW involved in the initial conceptualization of the manuscript. PT led the review of the literature, wrote the first draft, and involved in the visualization of concepts. FL and ZZ collected important background information and provided assistance for data acquisition. YC, XW, PT, FL, and ZZ provided revisions and additional conceptual input to the manuscript. All authors contributed to the article and approved the submitted version.

## Conflict of Interest

YC, XW, FL, and ZZ were employed by the Wuzhoufeng Agricultural Science and Technology Co., Ltd. The remaining author declares that the research was conducted in the absence of any commercial or financial relationships that could be construed as a potential conflict of interest.

## Publisher’s Note

All claims expressed in this article are solely those of the authors and do not necessarily represent those of their affiliated organizations, or those of the publisher, the editors and the reviewers. Any product that may be evaluated in this article, or claim that may be made by its manufacturer, is not guaranteed or endorsed by the publisher.
