# Supplementary material for: Aerospace Technology Improves Fermentation Potential of Microorganisms
Source: Front Microbiol. 2022 Apr 29;13:896556. doi: 10.3389/fmicb.2022.896556 (PMC9106405; doi:10.3389/fmicb.2022.896556)
Supplement: Supplementary file 1 [file Table_1.DOC]

Table S1. Space mutagenesis of microorganisms via a series of Shenzhou spacecrafts of China

| Spacecraft numbers | Date | Microorganisms | Results | References |
| --- | --- | --- | --- | --- |
| Shenzhou I | 20 November 1999 | *Streptomyces fradiae* | Tylosin, a type of macrolide antibiotic, increased by up to 91.5%；48 strains with 20 % higher titer | 1 |
| Shenzhou II | 10 January 2000 | *Escherichia coli* |  | 2 |
| Shenzhou III | 25 March 2002. | *Monascus purpureus* | higher productivity of Lovastatin | 3 |
|  |  | *Streptomyces fradiae* | Tylosin, a type of macrolide antibiotic, increased by up to 91.5%；48 strains with 20 % higher titer | 1 |
|  |  | *Bacillus*subtilis*,* Saccharomyces*cerevisiae,* Candida zemplinina | The application of bacillus, yeast and Hanoi white koji after space mutation breeding in the production of sesame-flavor liquor can reduce the amount of koji used, shorten the fermentation period and improve product quality and appearance (the appearance is light brown, the middle is dark brown, the cross section is neat. The color of the core is dark). The flavor of the curd sauce is strong. | 4 |
| Shenzhou IV | 30 December 2002 | *Streptomyces fradiae* | tylosin, a type of macrolide antibiotic, increased by up to 91.5%；48 strains with 20 % higher titer | 1 |
|  |  | *56 species of microorganism* |  | 2 |
| Shenzhou VI | 12 October 2005 | *No microorganism strains* |  |  |
| Shenzhou VII | 25 September 2008 | *Citrobacter freundii* | A mutant with higher violacein productivity more than 143.8%, which may be caused by the disturbance of gene network | 5 |
|  |  |  |  |  |
| Shenzhou VIII | 1 November 2011 | *Engineered bacterial strains* | Five mutant engineered bacterial strains showed a significantly higher production of recombinant human interferon a1b and one strain with the antibiotics activity up to 3-fold. | 6 |
|  |  | *Bacillus amyloliquefaciens, Enterococcus faecalis, Bacillus licheniformis, Staphylococcus aureus, Stenotrophomonas maltophilia, Burkholderia cepacia, Serratia marcescens, Acinetobacter lwoffii, Staphylococcus epidermidis, Canidia albicans, Klebsiella pneumoniae, Pseudomonas aeruginosa, Enterococcus faecium, Bacillus cereus and*  *Escherichia coli* | This revealed that effects were mainly seen in changes to bacterial invasion, antibiotic resistance and environmental adaptation. The mechanisms may be caused by various changes involving the genome, transcriptome, proteome and metabolome. | 7 |
|  |  | *Actinomycetes species, yeast, acetic acid bacteria, lactic acid bacteria, and Aspergillus species* | In this experiment, the changes in the flora of microorganisms before and after spaceborne were studied. The results showed that the number of actinomycetes in Daqu decreased significantly; the number of yeast, acetic acid bacteria and lactic acid bacteria decreased; the number of Aspergillus increased more. These changes are very beneficial to the formation of salt-free solids in vinegar, which can greatly improve the quality of brewed products. | 8 |
| Shenzhou IX | 16 June 2012 | *Tetrodotoxin strains* | The mutant tetrodotoxin strains via spatial mutagenesis can be used industrial production of toxins. After purification, they are mainly used for detoxification to effectively reduce the relapse rate of addicts. | 9 |
|  |  | *Streptomyces silaceus* | Streptomycetes silaceus carried on Shenzhou 9 spaceship and space microbial fertilizer prepared from *Streptomycetes silaceus*. | CN103160454B |
|  |  | *Actinomycetes species, yeast, acetic acid bacteria, lactic acid bacteria, and Aspergillus species* | In this experiment, the changes in the flora of microorganisms before and after spaceborne were studied. The results showed that the number of actinomycetes in Daqu decreased significantly; the number of yeast, acetic acid bacteria and lactic acid bacteria decreased; the number of Aspergillus increased more. These changes are very beneficial to the formation of salt-free solids in vinegar, which can greatly improve the quality of brewed products. | 8 |
| Shenzhou X | 11 June 2013 | *Lysobacter enzymogenes* | An increase in the production of the endoproteinase Lys-C of up to 40.2%, with perfect stability | 10 |
|  |  | Staphylococcus aureus | 10 potential deletion regions and 2 potential insertion regions were identified in the mutants with appearing more fragile than other strains. | 11 |
|  |  | *Actinomycetes species, yeast, acetic acid bacteria, lactic acid bacteria, and Aspergillus species* | In this experiment, the changes in the flora of microorganisms before and after spaceborne were studied. The results showed that the number of actinomycetes in Daqu decreased significantly; the number of yeast, acetic acid bacteria and lactic acid bacteria decreased; the number of Aspergillus increased more. These changes are very beneficial to the formation of salt-free solids in vinegar, which can greatly improve the quality of brewed products. | 8 |
| Shenzhou XI | 17 October 2016 | *Acinetobacter baumannii* | The biofilm formation ability of the flight strain was decreased after 33 days of spaceflight | 12 |
|  |  | *Sphingomonas paucimobilis* | A strain isolated from the Chinese spacecraft Shenzhou X is with 3,864 protein-coding and 50 RNA genes. | 13 |
|  |  | *Lactobacillus plantarum GS18* | Results showed that the isolate *L. plantarum* SS18–50 had the strongest probiotic properties with no mutation in 16S rRNA sequence compared to the wild type. Specifically, *L. plantarum* SS18–50 had good milk fermentation performance. The viscosity of fermented milk, acid tolerance, and bile salt tolerance were increased by approximately 10%, 8%, and 9%, respectively (*p* < .05). The antibacterial activity (*Escherichia Coli, Salmonella Typhimurium, and Listeria Monocytogenes*) was also increased significantly compared to the wild type (*p* < .05). | 14 |
|  |  | *Actinomycetes species, yeast, acetic acid bacteria, lactic acid bacteria, and Aspergillus species* | In this experiment, the changes in the flora of microorganisms before and after spaceborne were studied. The results showed that the number of actinomycetes in Daqu decreased significantly; the number of yeast, acetic acid bacteria and lactic acid bacteria decreased; the number of Aspergillus increased more. These changes are very beneficial to the formation of salt-free solids in vinegar, which can greatly improve the quality of brewed products. | 8 |
| Shenzhou XII | 17 June 2021 | *No reported yet* |  |  |
| Shenzhou XIII | 16 October 2021 | *No reported yet* |  |  |
| International Space Station (ISS) | 2012 | *Bacillus subtilis 168.* | After exposed to space for 1.5 years, mutations isolated from flight and parallel mission ground reference (MGR) samples were exclusively localized to Cluster I. The 21 RifR mutations isolated from the flight experiment showed all a C to T transition and were all localized to one hotspot: H482Y. In mutants isolated from the MGR, the spectrum was wider with predicted amino acid changes at residues Q469K/L/R, H482D/P/R/Y, and S487L. | 15 |
| US Space Shuttle STS-80 | 2002 | *Streptomyces plicatus* | The space flight reduced the cell numbers in CFU/mL of *S. plicatus* and increased the productivity of actinomycin D | 16 |
| Russian space station |  | *Saccharomyces cerevisiae* | The deletion of the ribosomal protein gene in the yeast *Saccharomyces cerevisiae* was detected after flight on the Russian space station, suggesting that space radiation containing high-linear energy transfer causing deletion-type mutants | 17 |
| Japanese Space Station KIBO | 2021 | *Aspergillus sydowii, Penicillium palitans, and Rhodotorula mucilaginosa* | The fungi are still increasing and expanding over time |  |
| ISS, BRIC-18 and BRIC-21 |  | *Bacillus subtilis* | Nucleotide sequencing of the Rif^R^ regions of the *rpoB* gene from rifampicin resistance  Rif^R^ mutants revealed dramatic differences in the spectrum of mutations between flight (FL) and ground control (GC) samples, including two newly discovered *rpoB* alleles in the FL samples (Q137R and L489S). | 18 |
| ISS, BRIC-21 and BRIC-23 | 2019 | *Bacillus subtilis* | Genes upregulated in flight samples notably included those involved in biofilm formation, biotin and arginine biosynthesis, siderophores, manganese transport, toxin production and resistance, and sporulation inhibition. | 19 |
| European Space EXPOSE-E | 2012 | *Bacillus subtilis* | The highly UV-resistant strain of *B. subtilis* was induced. | 20 |
| ISS, UK | 2021 | ***Bacillus pumilus****SAFR-032* | Genomic variants was found and m6A methylation increased in the mutant strains. | 21 |
| Mars Simulation Chamber, USA | 2019 | *Bacillus subtilis* | *Bacillus subtilis* spores were able to survive for at least a limited time in a simulated Martian environment, both with or without solar UV radiation. |  |
| ISS, USA | 2018 | *Aspergillus niger* | The ISS isolate exhibited an increased rate of growth and pigment distribution compared to a terrestrial strain. | 22 |
| NASA's BioSentinel mission | 2020 | *Saccharomyces cerevisiae* | Long-term yeast cell viability is maximized when cells are air-dried in trehalose solution and stored in a low-relative humidity and low-temperature environment and that dried yeast is sensitive to low doses of deep space-relevant ionizing radiation under these conditions. | 23 |
| ISS, EXPOSE-R2 | 2014 | *Circinaria gyrosa* | There was DNA damage in lichen exposed to harsh space and Mars-like environmental conditions, with ultraviolet radiation combined with space vacuum causing the most damage. | 24 |

**Figure preparation**

Figures 1 and 2 were prepared with the figures from the following websites with modification.

https://coquitlamwx.wordpress.com/2018/09/19/atsc-201-2-electromagnetic-radiation-from-the-sun-to-the-earth/

https://medium.com/predict/the-mysterious-source-of-extragalactic-cosmic-rays-2e649fed4130

https://www.nasaspaceflight.com/2021/06/shenzhou-12-new-chinese-station/

https://www.papertrell.com/apps/preview/The-Handy-Science-Answer-Book/Handy%20Answer%20book/What-is-the-difference-between-zero-gravity-and-microgravity/001137021/content/SC/52cb004082fad14abfa5c2e0_default.html

https://watchers.news/2015/06/09/cosmic-solar-radiation-as-the-cause-of-earthquakes-and-volcanic-eruptions/

https://pakistanweatherportal.com/2012/03/08/severe-solar-storm-striking-earth/

https://www.theweek.in/news/sci-tech/2019/04/01/New-insight-into-how-Suns-powerful-magnetic-field-effects-Earth.html

https://www.123rf.com/photo_96101433_stock-illustration-high-energy-particles-explosion-3d-illustration.html

https://svs.gsfc.nasa.gov/4539

https://www.nature.com/scitable/topicpage/yeast-fermentation-and-the-making-of-beer-14372813/

https://firstwefeast.com/drink/2014/07/infographic-what-gives-beer-its-flavor

https://www.morebeer.com/articles/focus_on_beer_flavor

https://beerandbrewing.com/actual-beer-color-vs-predicted-color/

https://hubblesite.org/contents/articles/the-electromagnetic-spectrum

https://en.wikipedia.org/wiki/Alpha_decay

**References**

1. Fang X-M, Zhao Z-J, Gu H-K. A study on space mutation of Streptomyces fradiae. *Hang Tian yi xue yu yi xue Gong Cheng= Space Medicine & Medical Engineering*. 2005;18(2):121-125.

2. Pei W, Hu W, Chai Z, Zhou G. Current status of space radiobiological studies in China. *Life Sciences in Space Research*. 2019;22:1-7.

3. Yin H, Xie S, Zhang G, Xie S. Effect of space flight on yield of Monascus purpureus. *Hang Tian yi xue yu yi xue Gong Cheng= Space Medicine & Medical Engineering*. 2003;16(5):374-376.

4. Bin Z, Mingyang Z, Jinhua W, Tao Y. Research on Space Breeding of Brewing Microorganisms and Its Application in Production (Ⅲ) Application of Space Bacteria in Sesame Flavor Liquor in Production. . *Winemaking*. 2015;2:21- 27.

5. Jiang P, Zhang R, Wang H, Xiao S, Yang C, Xing X. Outer space mutagenesis of violacein-producing strain and screening of mutants with high violacein productivity. *J Chem Ind Eng*. 2010;61:455-461.

6. Wang J, Liu C, Liu J, et al. Space mutagenesis of genetically engineered bacteria expressing recombinant human interferon α1b and screening of higher yielding strains. *World Journal of Microbiology and Biotechnology*. 2014;30(3):943-949.

7. Su L, Chang D, Liu C. The development of space microbiology in the future: the value and significance of space microbiology research. *Future Microbiology*. 2013;8(1):5-8.

8. Zongzhou Z, Yaping X. The impact of spaceborne on the microflora of Daqu. *Food science*. 2009;3:214-216.

9. Mulkey TJ. 2010 Annual Meeting-ABSTRACTS. *Gravitational and Space Research*. 2010;24(1)

10. Liu C. The theory and application of space microbiology: China's experiences in space experiments and beyond. *Environmental microbiology*. 2017;19(2):426-433.

11. Guo J, Han N, Zhang Y, et al. Use of genome sequencing to assess nucleotide structure variation of Staphylococcus aureus strains cultured in spaceflight on Shenzhou-X, under simulated microgravity and on the ground. *Microbiological Research*. 2015;170:61-68.

12. Zhao X, Yu Y, Zhang X, et al. Decreased biofilm formation ability of Acinetobacter baumannii after spaceflight on China's Shenzhou 11 spacecraft. *MicrobiologyOpen*. 2019;8(6):e00763.

13. Pan L, Zhou H, Li J, et al. Draft genome sequence of Sphingomonas paucimobilis strain LCT-SP1 isolated from the Shenzhou X spacecraft of China. *Standards in Genomic Sciences*. 2016;11(1):1-7.

14. Wang D, Zhang T, Ye H, Hao H, Zhang H, Zhao C. In vitro probiotic screening and evaluation of space‐induced mutant Lactobacillus plantarum. *Food Science & Nutrition*. 2020;8(11):6031-6036.

15. Nicholson WL, Moeller tPT, Ralf, Horneck G. Transcriptomic responses of germinating Bacillus subtilis spores exposed to 1.5 years of space and simulated martian conditions on the EXPOSE-E experiment PROTECT. *Astrobiology*. 2012;12(5):469-486.

16. Lam K, Gustavson D, Pirnik D, et al. The effect of space flight on the production of actinomycin D by Streptomyces plicatus. *Journal of Industrial Microbiology and Biotechnology*. 2002;29(6):299-302.

17. Fukuda T, Fukuda K, Takahashi A, et al. Analysis of deletion mutations of the rpsL gene in the yeast Saccharomyces cerevisiae detected after long-term flight on the Russian space station Mir. *Mutation Research/Genetic Toxicology and Environmental Mutagenesis*. 2000;470(2):125-132.

18. Fajardo-Cavazos P, Leehan JD, Nicholson WL. Alterations in the spectrum of spontaneous rifampicin-resistance mutations in the Bacillus subtilis rpoB gene after cultivation in the human spaceflight environment. *Frontiers in Microbiology*. 2018;9:192.

19. Morrison MD, Fajardo-Cavazos P, Nicholson WL. Comparison of Bacillus subtilis transcriptome profiles from two separate missions to the International Space Station. *npj Microgravity*. 2019;5(1):1-11.

20. Wassmann M, Moeller R, Rabbow E, et al. Survival of spores of the UV-resistant Bacillus subtilis strain MW01 after exposure to low-earth orbit and simulated martian conditions: data from the space experiment ADAPT on EXPOSE-E. *Astrobiology*. 2012;12(5):498-507.

21. Waters SM, Ledford SM, Wacker A, et al. Long-read sequencing reveals increased occurrence of genomic variants and adenosine methylation in Bacillus pumilus SAFR-032 after long-duration flight exposure onboard the International Space Station. *International Journal of Astrobiology*. 2021;20(6):435-444.

22. Romsdahl J, Blachowicz A, Chiang AJ, et al. Characterization of Aspergillus niger isolated from the international space station. *MSystems*. 2018;3(5):e00112-18.

23. Santa Maria SR, Marina DB, Massaro Tieze S, Liddell LC, Bhattacharya S. BioSentinel: long-term Saccharomyces cerevisiae preservation for a deep space biosensor mission. *Astrobiology*. 2020;

24. de la Torre Noetzel R, Ortega Garcia MV, Miller AZ, et al. Lichen vitality after a space flight on board the EXPOSE-R2 facility outside the international space station: results of the biology and mars experiment. *Astrobiology*. 2020;20(5):583-600.
